# Peptide Array X-Linking (PAX): A New Peptide-Protein Identification Approach

**DOI:** 10.1371/journal.pone.0037035

**Published:** 2012-05-14

**Authors:** Hirokazu Okada, Akiyoshi Uezu, Erik J. Soderblom, M. Arthur Moseley, Frank B. Gertler, Scott H. Soderling

**Affiliations:** 1 Department of Cell Biology, Duke University Medical School, Durham, North Carolina, United States of America; 2 Institute for Genome Science & Policy Proteomics Core Facility, Duke University Medical School, Durham, North Carolina, United States of America; 3 Koch Institute for Integrative Cancer Research at MIT, Massachusetts Institute of Technology, Cambridge, Massachusetts, United States of America; Karolinska Institutet, Sweden

## Abstract

Many protein interaction domains bind short peptides based on canonical sequence consensus motifs. Here we report the development of a peptide array-based proteomics tool to identify proteins directly interacting with ligand peptides from cell lysates. Array-formatted bait peptides containing an amino acid-derived cross-linker are photo-induced to crosslink with interacting proteins from lysates of interest. Indirect associations are removed by high stringency washes under denaturing conditions. Covalently trapped proteins are subsequently identified by LC-MS/MS and screened by cluster analysis and domain scanning. We apply this methodology to peptides with different proline-containing consensus sequences and show successful identifications from brain lysates of known and novel proteins containing polyproline motif-binding domains such as EH, EVH1, SH3, WW domains. These results suggest the capacity of arrayed peptide ligands to capture and subsequently identify proteins by mass spectrometry is relatively broad and robust. Additionally, the approach is rapid and applicable to cell or tissue fractions from any source, making the approach a flexible tool for initial protein-protein interaction discovery.

## Introduction

Many regulatory proteins that govern cellular signaling events adopt modular structures consisting of multiple domains [1], [2]. These domains form independent, three-dimensional structures to exert specialized functions within the cell [3]. The functionality of these domains can be primarily divided into two major roles: interactive and enzymatic. Interactive domains bind to proteins, lipids or nucleotides and control the residing protein's activity, intracellular localization and stability [1]. Among the interactive domains, many domains bind to short peptide ligands [4]. Characterization of the binding specificity has determined consensus motifs in the target peptide sequences for many of these domains [4]. Thus, it is now to some extent possible to anticipate the class of domains that may bind to a certain peptide with a consensus motif. However, it is still impracticable to predict individual domains/proteins that actually interact with the peptide in the cell since the specificity for the individual domains is determined by amino acids not involved in the consensus motifs. For instance, actin cytoskeletal regulatory proteins such as formin-family proteins or Arp2/3-dependent actin nucleation promoting factors (NPF) often have poly-proline sequences [5], [6], but the proteins that interact with these sequences are largely unknown. Although it is expected that the poly-proline sequences may interact with proteins with EH, EVH1, SH3, WW domains [4], [7], [8], [9], time consuming approaches are still required to identify the specific proteins involved.

One of primary methodologies to identify signaling complexes based on protein interactions is affinity purification (AP) followed by mass spectrometry (MS) [10], [11]. In this case, immobilized bait proteins are incubated with cell/tissue lysates and, after extensive washes to remove non-specific interactors, the binding partners are identified by MS analysis. However, this approach has some disadvantages: the extensive washing disrupts weak or transient interactions, and no information is provided on which proteins are direct interactors with the bait among the identified proteins. Another strategy is the yeast two-hybrid (YTH) screen. This method identifies direct interactions because the YTH is based on bimolecular interactions to activate reporter gene transcription [12]. There, however, are some limitations: the interactions have to occur in the yeast nucleus, post-translational modifications on the bait peptides/proteins that may occur in mammalian cells such as phosphorylation are unlikely to be recapitulated within yeast, and false positive rates can be high due to spurious transcriptional activation by bait sequences. Moreover, both AP-MS and YTH approaches are difficult to perform with large numbers of bait targets due to the extensive optimization for individual proteins.

To circumvent several of these problems, we have developed a peptide array-based cross-linking strategy. Here we report that our strategy identifies both previously known and novel interactions for peptides with different consensus motifs from brain tissue lysates. We also apply the method to a poly-proline sequence of Wiskott-Aldrich syndrome protein family member 1 (WAVE1), a member of WAVE/WASP family NPF proteins, and demonstrate the identification of novel interacting proteins.

## Results

### PAX–Captured Proteins Are MS Identifiable

**Figure 1:**
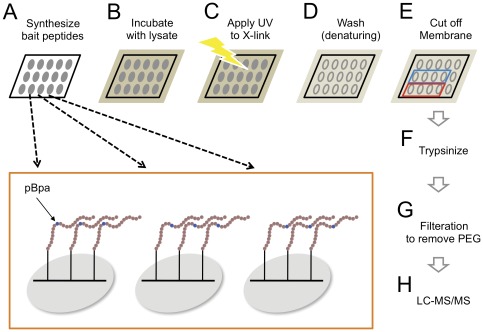
Experimental outline of PAX methodology. (**A**) Arrays of bait peptides are synthesized onto PEG-based membrane supports. The photo-activatable amino acid cross-linker (pBpa) is incorporated into the bait peptides so that each peptide spot has pBpa at a different position in the sequence (see inset schematic). (**B**, **C**) The membrane is incubated with cell lysate and subjected to 350–365 nm light to cross-link with interacting proteins. (**D**) The indirect and non-specific interactors are removed by high stringent, denaturing washes. (**E**, **F**) Each strip of the bait peptide spots is cut off the membrane, further chopped into small pieces, and trypsinized. (**G**, **H**) The samples are filtered to remove PEG and subjected to LC-MS/MS analysis to identify the photo-trapped proteins.

Benzophenone photoprobes, which covalently couple to unreactive C-H bonds upon exposure to 350–360 nm light, have been widely used for biochemical characterization of macromolecules since the 1980s [13], [14]. More recently the benzophenone phenylalanine derivative, *p*-benzoyl-L-phenylalanine (pBpa) has been successfully translated into proteins of interest within cells, enabling the photo-induced crosslinking of protein-protein interactions as they occur in situ [15], [16], [17], [18], [19]. We have used this approach to purify and discover new interactions by incorporating pBpa into protein binding domains expressed within mammalian cells [20]. The success of this approach prompted us to wonder if the reverse strategy, utilizing peptides containing pBpa, could also be useful for the rapid identification of protein interactions from cell lysates. The primary obstacle to this approach is that pBpa must be positionally placed within the peptide such that it is close enough to capture a binding protein, but not within the binding site so as to not disrupt the relevant interaction. This requirement creates a problem, however, since permissive positions within a putative peptide ligand are unknown for uncharacterized interactions that are the target of protein interaction discovery approaches. This problem could be circumvented, however, if the peptide ligand is represented as an array of peptide sequences, with pBpa placed at each possible position within the peptide (Figure 1A and inset). In this format, potential interactions with the peptide may be covalently captured by those peptides in which pBpa is at a permissive position for binding and crosslinking, without prior knowledge of which site is optimal. Previously we have used the SPOT technology to synthesize arrays of peptide sequences on PEG derivatized cellulose membranes [20], [21], [22]. 384 peptides can be synthesized on a membrane within a 24×16 spot format, and up to 4 membranes can be processed at a time. We hypothesized that pBpa containing peptides arrayed in this format, incubated with cell or tissue lysates, and exposed to UV light in the 350–360 nm range might provide a convenient and rapid approach to phototrap peptide-protein interactions. After incubation with cell/tissue lysates of interest and photo-induced covalent trapping of binding proteins (Figure 1B–C), the peptide array can be extensively washed under denaturing conditions to aid the removal of indirect and non-specific interactors (Figure 1D). Peptides with bound proteins would then be trypsin digested and analyzed by mass spectrometry (Figure 1E–H).

**Figure 2:**
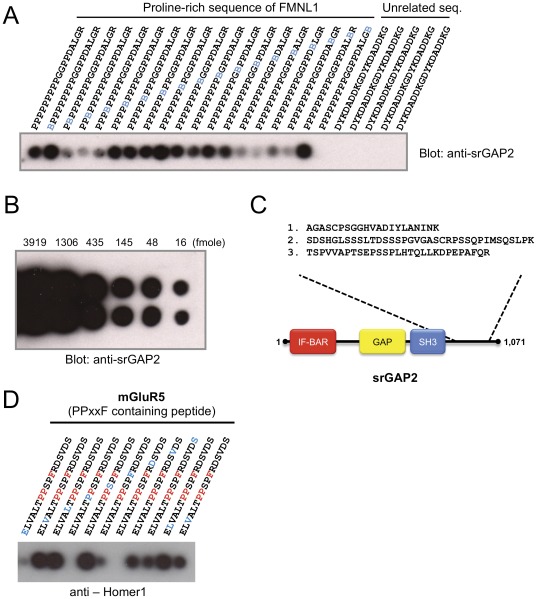
PAX-captured proteins are MS identifiable. (**A**) PAX captures a known interacting protein following over-expression in FreeStyle 293 cells. An array of peptides consisting of a proline-rich sequence of FMNL1 and a control sequence were incubated with FreeStyle 293 cell lysate overexpressing srGAP2, photo-crosslinked and washed at high stringency. Immunoblotting with srGAP2 antibody detected phototrapping of srGAP2 by PAX. pBpa in the bait peptides is indicated in blue. (**B**) Comparative immunoblotting of srGAP2 antigen dot-blot. Different amounts of srGAP2 were spotted onto nitrocellulose membrane and simultaneously immunoblotted with srGAP2 antibody. (**C**) Identification of srGAP2 by mass spectrometry. The five spots with the highest amount of crosslinked srGAP2 from a duplicate blot of (A) were subjected to MS/MS. 3 unique peptides (underlined) covering 8% of the entire sequence of srGAP2 protein were identified. (**D**) PAX captures a known interacting protein from mouse brain lysate. Peptide array comprising mGluR5 sequences with a PPXXF motif was incubated with mouse brain lysate, photo-crosslinked and subjected to high stringency washes. Immunoblotting with Homer1 antibody indicates photo-trapping of Homer1 by PAX. pBpa in the bait peptides is indicated in blue.

We first investigated if proteins photo-trapped to arrayed bait peptides synthesized on cellulose membrane are identifiable by mass spectrometry. The primary concern was whether enough bound protein could be photo-trapped within the peptide spots to be subsequently detected by mass spectrometry. For nanoscale capillary UPLC columns typically used in LC-MS experiments, optimal column loads between 500 ng and 1 ug of total protein digest are desired. Since this direct-interactor only array crosslinking experiment results in a much simplier composition of proteins, column loads as low 100 ng would yield an appropriate column load because the moles of each protein per amount of input material would be significantly higher. As a test case, we ectopically expressed SLIT-ROBO Rho GTPase-activating protein 2 (srGAP2) in FreeStyle 293 suspension cells whose SH3 domain is known to interact with a proline-rich sequence within Formin-like protein 1 (FMNL1) [22]. Cell lysate from transfected cells was incubated with FMNL1 poly-proline peptide arrays with each of the peptide spots containing pBpa at a distinct position (Fig. 2A). The membrane was subjected to UV light (365 nm) to induce cross-linking and then washed extensively with a harsh denaturing solution (0.1% Tween-20, 1% SDS, pH2.5, 95°C: See Method for more details). Immunoblotting was then performed using an anti-srGAP2 antibody, demonstrating that srGAP2 was photo-trapped to the peptide spots on the membrane (Fig. 2A). The amount of the captured srGAP2 varied, dependent on the positions of pBpa within the bait peptide, indicating which amino acids are critical for the interaction. Unexpectedly, the peptide without pBpa also showed positive signal, suggesting this interaction could survive the high stringent washes to a lesser extent. Unrelated peptides did not capture any trace of srGAP2, demonstrating the bound srGAP2 that was detected was specific.

To estimate the amounts of the captured srGAP2, we dot blotted known concentrations of the srGAP2 antigen onto nitrocelluse (Fig. 2B). The dot blot membrane was incubated with anti-srGAP2 antibody solution together with the PAX membrane mentioned above. Comparison of the signal strength revealed that the strongest signals of the PAX blot captured an estimated 10–20 femtomoles of srGAP2, suggesting enough material may be captured for MS analysis. This possibility was tested with the five strongest peptide spots (equivalent to 50–100 femto moles) from a duplicate membrane strip, which were cut into small pieces, gathered in a tube, trypsinized, filtered through a spin column to remove contaminating PEG, and applied to LC-MS/MS (Fig. 1). MS analysis identified three unique peptides corresponding to the srGAP2 protein, confirming prey proteins can be captured from cell lysates for MS identification (Fig. 2C).

We next tested if PAX methodology could photo-trap endogenous proteins from brain lysates. Peptides from Metabotropic glutamate receptor 5 (mGluR5) that are known to bind the class II (Homer/Vesl proteins) EVH1 domain of a postsynaptic density scaffolding protein Homer1 [23] were incubated with brain lysates from adult mice and subjected to PAX. pBpa position-dependent interactions were observed by immunoblotting using anti-Homer1 antibody (Fig. 2D). As expected, the replacement of the amino acids of the consensus motif with pBpa abolished the interaction. Interestingly, the replacement of acidic amino acids (Glu and Asp) outside the motif also abolished the interaction, revealing additional amino acids for the interaction that are indispensible.

### PAX-MS Identifies Known and Novel Interactions from Brain Lysates

To examine the applicability of PAX as a proteomics tool to identify peptide-protein interactions, we applied PAX to several peptide ligands with proline-containing motifs, including Son of sevenless homolog 1 (SOS1), Stonin2, Epsin1 and mGluR5, each of which has previously known binding partners (Table 1) [23], [24], [25], [26], [27], [28], [29]. A negative control peptide without homology (no more than 50% sequence identity to any mammalian sequences) or proline-containing binding motifs was used. All the bait peptides were synthesized in an array format on the same membrane. Each bait consisted of 12 peptide spots, and each of these peptides contained pBpa at different positions. The membrane was incubated with mouse brain lysate made from two adult brains, exposed to UV light to photo-trap interacting proteins, washed at high stringency and cut off into individual bait strips. Each strip was then trypsinized and subjected to MS analysis to identify the photo-trapped proteins. This procedure was performed in duplicate and identified a total of 198 proteins. To minimize false positives, we used high stringency criteria [0.2% protein and 4.9% peptide false discovery rates] for this MS identification. In protein interaction studies, elimination of non-specific interactions is critical. To extract high confidence interactions, we performed two complimentary data analyses: (1) identification of selective bait interactors, and (2) protein interaction domain filtering.

**Table 1:** List of Bait Peptides and the Identified Proteins.

Bait			Prey	
Protein	Peptide	Binding motifs (found in bait)	Domains	Identified proteins★
Control	AYGDLPFYVRSDGLRSHF	None	None	
SOS1	VP**PP**V**PP**RRRPESAPAES	PXXP	SH3	*Grb2, Itsn1*, ***Pacsin1, Sh3kbp1, Cd2ap***, Amph
Stonin2	PWRAT**NPF**LNETLQ	NPF	EH	*Eps15l1, Itsn1*, Ehd3
Epsin1	GAKAS**NPF**L**P**SGAP	NPF PXXP	EH SH3	*Eps15l1*, Itsn1, Amph
mGluR5	ELVALT**PP**S**PF**RDSVDS	PPXXF PXXP	EVH1 (class II) SH3	*Homer1*, ***Homer3***
WAVE1	PQGEVQG**LPPPPPPPPLP**	PXXP PPLP (F/L)PPPP	SH3 WW EVH1 (class I)	Vasp, Gas7, Enah, Amph

★ Proteins in italics were previously known to bind to the bait peptides and reproduced in the study. The interactions between proteins in bold italics and the bait proteins were identified in the bioinformatics study.

**Figure 3:**
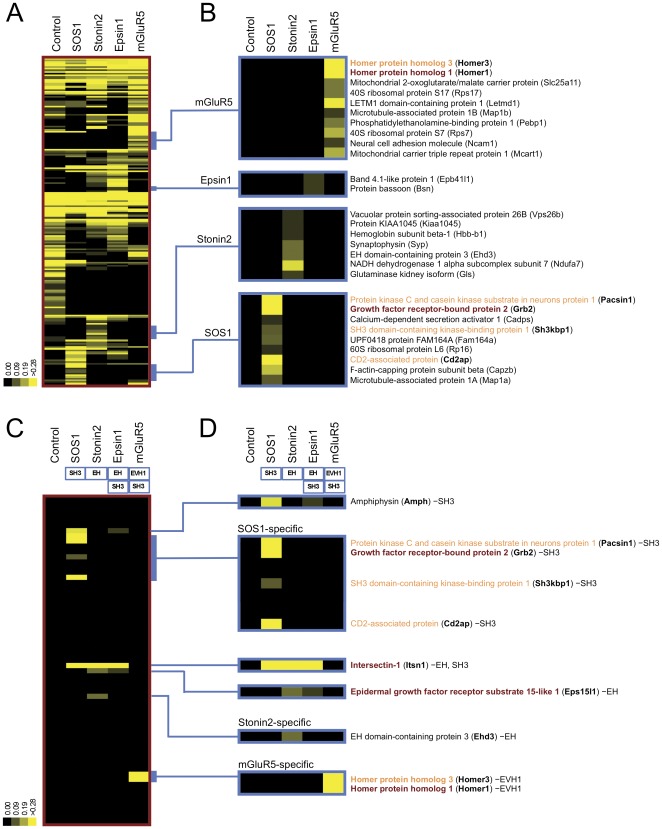
PAX successfully identifies interacting proteins from tissue lysates. (**A**) Cluster analysis of MS/MS-identified proteins. Candidate interactors with the bait peptides from SOS1, Stonin2, Epsin1 and mGluR5 were hierarchically clustered using unbiased Pearson correlation of the mean normalized spectral counts. Protein clusters of single bait interactors are indicated with blue bars. (**B**) Identification of selective bait interactors. Single bait interactors identified in (A) are exhibited. Proteins in brown are known to bind to the corresponding bait peptides. Bioinformatics analysis revealed that proteins in orange were previously shown to interact with the bait proteins. (**C**) Protein interaction domain filtering. Proteins that showed association to the control bait peptide were eliminated from the MS-identified proteins, and the rest was subjected to cluster analysis. Subsequently, the proteins that contain domains anticipated to interact with consensus binding motifs found in bait peptides were selected as high confidence interactors. (**D**) Identification of the interactors with anticipated peptide recognition domains. Proteins were colored as explained in (B).

First, to decode the association pattern of the MS-identified proteins for each bait peptide, we performed cluster analysis using Pearson correlation of the mean normalized spectral counts, which are MS measurements that reflect the relative abundance of the proteins in the PAX sample (Fig. 3A). In this analysis, we focused only on the proteins that were associated with single bait peptides. These interactions are of higher confidence because the interactions with the other bait peptides function as negative controls and proteins interacting with all peptides are likely to be non-specific. The cluster analysis produced 4 protein clusters that showed a specific association to each bait peptide for mGluR5, Epsin1, Stonin2 and SOS1 (Fig. 3B). The mGluR5-specific cluster contained Homer1, a known interactor of the mGluR5 peptide (Table 1). The SOS1-specific cluster also contained GRB2, a known interactor of the SOS1 peptide. We next performed bioinformatics searches to see if there are any other known interactions among the bait proteins and the corresponding protein clusters (see the Method section). This search identified another known interactor in the mGluR5 protein cluster (Homer3) and 3 known interactors (Pacsin1, Sh3kbp1, Cd2ap) in the SOS1-specific protein cluster. Thus, single bait interaction clusters identified previously known interactions including our “positive controls” from brain lysates. The rest of the interactors in the clusters are previously-unidentified binding proteins and to verify these interactions, additional assays such as co-immunoprecipitation or co-localization experiments are required.

Clustering analysis is useful to identify higher confidence interactions but has an obvious limitation; proteins that contain domains, which may interact with related bait sequences are excluded. An advantage to using peptide sequences as baits is that it is possible to predict the type of protein interaction domain that may bind to it. To capitalize on this, we utilized consensus binding motifs found in the bait peptides to predict domains that should be found within legitimate binding proteins. Table 1 describes consensus binding motifs identified in the bait peptides and the domains that are expected to bind these motifs. To filter the interaction data by expected domains, we eliminated all the proteins that showed association to the control peptide and performed cluster analysis. We then filtered the data for proteins containing the expected interaction domains. Thus all the proteins that remained indicate high confidence interactions predicted to bind to each bait peptide based on its primary sequence and the corresponding interaction domains (Fig. 3C). This domain filtering identified all the previously known interactions identified by the cluster analysis, but also found potentially meaningful new interactions that had previously been discarded (Fig. 3D). For example, the domain filtering identified 3 other proteins that interacted with multiple baits. Intersectin-1 (Itsn1) was positive for SOS1, Stonin2 and Epsin1 because of its SH3 domain and EH domain. The interactions with the SOS1 and Stonin2 peptides were previously identified (Table 1), and thus the interactions with Epsin1 are newly identified by the domain filtering. Epidermal growth factor receptor substrate 15-like 1 (Eps15l1) remained positive for Stonin2 and Epsin1 because of its EH domain. These interactions were also previously identified (Table 1). Amphiphysin (Amph) was positive for SOS1 and Epsin1 because of its SH3 domain. Amphiphysin2 (Amph2), highly homologous to Amph1, is known to bind to the proline-rich region of SOS1 [30], indicating this newly identified interaction between SOS1 and Amph1 is likely. Epsin1 was identified as a novel interactor. Thus the domain filtering identified most of the expected and known interactions. Combining the two analyses, we were able to select 10 high confidence interacting proteins (14 high confidence interactions) out of 198 MS-identified interactors (544 possible interactions).

### PAX-MS Identifies Novel Interactions with WAVE1

**Figure 4:**
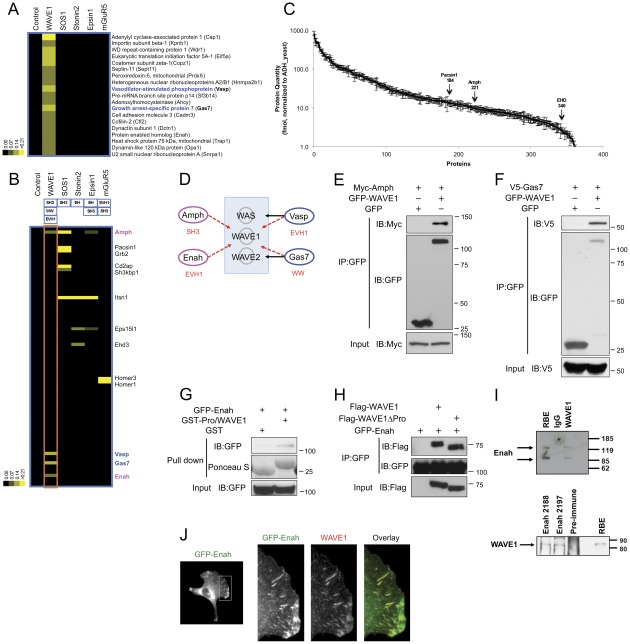
PAX identifies novel interacting proteins with WAVE1. (**A**) Identification of ligands for a WAVE1 derived peptide. MS/MS data for WAVE1 trapped proteins from brain lysate were combined with those for control, SOS1, Stonin2, Epsin1 and mGluR5 and subjected to cluster analysis as in Fig. 3A. Vasp and Gas7 are known to interact with other members of the WAVE/WASP family proteins. (**B**) Protein interaction domain filtering. Enah and Amph, in addition to Vasp and Gas7, were identified as WAVE1 peptide-interacting proteins. (**C**) Only 3 of the 13 proteins identified in (B) were identified as abundant proteins using label-free LC/MS/MS quantification of mouse brain lysates. (**D**) WAVE1 peptide interactome. PAX identified high confidence interactions (red dotted arrows) with WAVE1. The previously known interactions are indicated with black arrows. The WAVE1 selective binding proteins contain domains (shown in red) that are expected to interact with the WAVE1 polyproline peptide (see Table 1). (**E–F**) Co-immunoprecipitation assays with full-length WAVE1 and (E) Amph or (F) Gas7. (**G**) GST pulldown of Enah using the proline peptide bait of WAVE1. (H) Co-immunoprecipitation of GFP-Enah with full-length WAVE1 and WAVE1 lacking the bait proline peptide. (**I**) Physiological interaction between WAVE1 and Enah. Reciprocal co-immunoprecipitation experiments were performed using rat brain extract (RBE). The precipitates were analyzed by Western blotting. (**J**) Co-localization of WAVE1 and Enah in fibroblast cells. Immunostaining of endogenous WAVE1 showed co-localization with Enah in MV^D7^ cells expressing GFP-tagged Enah.

We next applied the PAX and subsequent data analyses to the proline-rich sequence of WAVE1, a member of the Arp2/3-dependent actin nucleation promoting factors, to identify novel interactions from mouse brain lysates. To apply the clustering analysis, we combined MS data for WAVE1 with the previous MS data obtained for SOS1, Stonin2, Epsin1 and mGluR5 to elucidate WAVE1-specific protein cluster (Fig. 4A). The subsequent bioinformatics analysis identified no previously known interactors with WAVE1 but 2 plausible interactors, Vasodilator-stimulated phosphoprotein (Vasp) and Growth arrest-specific protein 7 (Gas7), that both have been revealed to interact with highly homologous family members of WAVE1 (WAS and WAVE2).

We next performed the domain filtering (Fig. 4B) and identified a total of 13 proteins for the five different baits. We assessed the abundance of proteins from the input brain lysate over a range of approximately three orders of magnitude. Only three of the proteins were identified (Pacsin1, Amph, and EHD) (Fig. 4C), suggesting most of the interactions detected were from low abundance proteins within the lysate. In comparison, analysis of the negative control peptide interactions revealed the top 13 proteins, based on the percentage of total spectra, were all highly abundant proteins. Twelve of these ranked within the top 100 abundant proteins of the lysate (EF1A1: #46, TBA1A/B: #8, CN37: #75, CLH: #47, ACTB: #11, G3P: #5, KCRB: #14, NSF: #95, SYN2: #93, TBB3: #37, KPYM: #23, TBB2B/C: #7). For WAVE1, the same interactors (Vasp and Gas7) were identified in the domain filtering analysis (Fig. 4D). Both the proteins have one of the expected domains for the WAVE1 bait peptide, WW and the class I (Ena/Vasp proteins) EVH1 proline binding domains (Table 1). Domain filtering also identified 2 more novel interactors, Amphiphysin (AMPH) and Enabled (Enah). Interactions between the full-length proteins of WAVE1 and Amphiphysin (Fig. 4E) and Gas7 (Fig. 4F) were confirmed by co-expression and co-immunoprecipitation. Enah is a homolog of Vasp but no interactions with WAVE/WASP family proteins have been reported. We first determined whether the WAVE1 peptide initially used as a bait would be sufficient to pulldown Enah from cell extracts (Fig. 4G). GST fused WAVE1 proline peptide (GST-Pro/WAVE1) weakly interacted with GFP-Enah, whereas GST alone did not. To confirm and further characterize the interaction between WAVE1 and Enah, we performed co-immunoprecipitations with Flag-tagged full length WAVE1 or WAVE1 lacking the proline peptide used as bait (WAVE1 ΔPro) and GFP-Enah (Fig. 4H). WAVE1 and Enah readily co-precipitated when compared to negative control immunopreciptations. Surprisingly, Enah also co-precipitated with WAVE1 lacking the bait peptide sequence. These results suggest that while the WAVE1 bait peptide is sufficient to interact with Enah, other interactions also participate in the binding reaction. We have previously noted similar results with WRP, which also binds to the proline rich region of WAVE1 in addition to other secondary interactions [31]. Reciprocal co-immunoprecipitation experiments were next performed to determine if endogenous WAVE1 and Enah interact. The interaction between the endogenous WAVE1 and Enah in mouse brain extracts was validated (Fig. 4I). Ectopically expressed GFP-tagged Enah also showed co-localization with endogenous WAVE1 in mouse fibroblast cells, confirming their colocalization cells (Fig. 4J and [32]). Together these data demonstrate the PAX methodology and the data analyses can be a useful tool to rapidly identify novel peptide-protein interactions from cell or tissue lysates, including those involving lower abundance proteins.

## Discussion

In the study of peptide recognition domains, phage display in which diverse libraries of phage-derived peptide ligands are exploited to determine the binding affinity and specificity for a specific domain has been a powerful strategy [33]. The resulting consensus binding peptide sequences can be utilized to bioinformatically search for candidate ligand sequences in genomes and these candidate peptides can be validated as ligands in subsequent experiments [34], [35], [36]. This approach, however, is domain centric and usually is biased towards identifying a large number of candidate peptides for a limited number of domains.

In the present study, our goal was to identify interactions with peptide ligands from the whole proteome of selected lysates. To accomplish this we have developed a unique proteomics tool in which peptide arrays are used as baits to isolate interacting proteins from tissue lysates. We have demonstrated in a proof-of-principle manner the mass spectrometry-based identification of known and novel interacting proteins. This method allows for the rapid identification of direct protein-peptide associations, based on the covalent capturing of interactions via a photo-activatable unnatural amino acid cross-linker incorporated into bait peptides. The covalent fixation of direct interactors to the baits therefore made possible the simple and efficient removal of indirect associations by high stringency washes. In the present study, we have applied this PAX method to bait peptides with various proline-containing consensus motifs and demonstrated the MS-based identification of interactions. We have also shown how two independent data analyses: (1) cluster analyses to select single bait interactors and (2) domain filtering successfully identified both known and novel interactions.

The application of the methodology to the proline-rich sequence of WAVE1 revealed 4 high confidence interactions. One of the interactions between WAVE1 and Enah was further confirmed by additional assays. Enah localizes at the leading edge of lamellipodia and the tip of fillopodia, which are protrusion structures of the cell comprising highly branched or long and unbranched actin filaments, respectively. Enah has been shown to positively regulate filopodia formation in part by binding to and protecting the fast growing barbed ends of actin filaments [37]. The WAVE/WASP family proteins faciliate the formation of lamellipodia by initiating the Arp2/3-based branching of actin filaments. These branched filaments may form the foundation for subsequent filopodia formation, whose actin filaments emanate from the lamellipodial structure [6], [38]. A recent study has revealed that WAVE2 forms a protein complex with Arp2/3 and mDia2, a formin-family protein that initiates and elongates unbranched actin filaments by also binding to the barbed end of filaments [39]. Binding of WAVE2 inhibits mDia2-mediated filopodia formation, providing with an unexpected role for WAVE2 in the inhibition of filopodia initiation. Our finding suggests a new link between WAVE1 and the filopodia-regulatory protein Enah. The exact functional relationship remains to be resolved, but it is possible that Enah is released from WAVE1 to protect newly nucleated filaments from capping, allowing them to subsequently elongate.

The PAX methodology presented here has a several advantages. Multiple baits can be synthesized on a single membrane and these can be simultaneously incubated with a lysate to photo-trap interacting proteins, thus enabling rapid, high-throughput experiments for several peptide sequences. Photo activation-dependent cross-linking also enables the potential trapping of protein interactions within the context of various experimental schemes such as lysates from stimulus or inhibitor treated cells or tissue. Thus the experimental procedures can be flexibly designed according to the types of expected ligands for each peptide bait. PAX also has a broad potential utility; it is applicable to any cell or tissue lysates but can also be used to identify organelle or subcellular fraction-dependent protein interactions. The availability of a broad range of modified amino acids and the ability to incorporate them into synthetic bait peptides by SPOT technology should provide interesting future applications for PAX, such as the identification of post-translational modification-dependent protein interactions. It should be noted, however, that additional experimental validations are required, especially to ensure non-native interactions are not induced by the incorporation of unnatural amino acids. In summary, the PAX methodology developed in this study presents a versatile proteomics tool with a broad potential application for rapid peptide-protein interaction discovery.

## Materials and Methods

### Antibodies

Rabbit polyclonal srGAP2, Enah and WAVE1 antibody have been previously described [21], [22], [40]. Antibody against Homer1, was purchased from BD Transduction. Horseradish peroxidase (HRP)-conjugated and Alexa-labeled secondary antibodies were purchased from GE Healthcare and Invitrogen, respectively. GFP-trap agarose (ChromoTek) was used for anti-GFP immunoprecipitations. Anti-V5 epitope antibody was purchased from Invitrogen, Anti-Myc antibody clone 9E10 was purchased from Millipore.

### Plasmids

GFP-WAVE1, Flag-WAVE1, and Flag-WAVE1ΔPro were previously described [21]. GFP-Mena and was obtained from Frank Gertler (MIT) and Myc-Amph, was obtained from Tadaomi Takenawa (Kobe Univ., Japan) and Hiroyuki Nakanishi (Kumamoto Univ., Japan). Full length GAS7 cDNA (Accession BC001152) was purchased from Open Biosystems. GST-ProWAVE1 was constructed by cloning the coding sequence of WAVE1 encompassing the peptide sequence PQGEVQGLPPPPPPPPLP into the pGEX-4T1 vector (GE healthcare Bio-Sciences).

### Peptide Array Synthesis

Peptides (14–18 mer) were synthesized as previously described [21] using Auto-Spot Robot ASP 222 (INTAVIS AG, Köln, Germany) except that all the procedure was performed in the dark to avoid undesirable photoactivation of pBpa.

### Peptide Array X-linking (PAX)

For the PAX of srGAP2 to FMNL1 poly-proline peptide, the following method was used. A 500 µl culture of FreeStyle 293 cells (Invitrogen) was grown in FreeStyle 293 medium (Gibco) to the density of 1×10^6^ cells/ml. Transfection was done using 500 µg of full-length srGAP2 DNA as described previously [20]. After a 3-day incubation at 37°C, the cells were pelleted, lysed in 10 ml of ice-cold lysis buffer (25 mM HEPES pH7.4, 150 mM NaCl, 1 mM EDTA, 0.5% Triton X-100, 1 mM 4-(2-aminoethyl)benzenesulfonyl fluoride hydrochloride (AEBSF), 2 µg/ml Leupeptin/Pepstatin), homogenized with a Dounce homogenizer, and subjected to centrifugation at 17,200× g for 30 min. The supernatant was further clarified by ultracentrifugation at 100,000 g for 1 hour. The supernatant was kept as cell lysate. For the PAX of brain lysates, two frozen adult mouse brains (Pel-Freez Biologicals) were crushed into small pieces using pestle and mortar, resuspended in 20 ml of ice-cold lysis buffer, homogenized with a Dounce homogenizer, and subjected to centrifugation at 17,200× g for 30 min. The supernatant was kept as brain lysate.

The peptide array membrane shielded from light was incubated for 2 h in a blocking buffer (5% nonfat dry milk, 1% bovine serum albumin and 0.01% sodium azide in TBST (10 mM Tris pH7.5, 150 mM NaCl, 0.05% Tween-20)). After brief wash with TBST, the membrane was incubated with the above brain lysate overnight at 4°C and induced to cross-link under UV (365 nm) for I h. Following a brief wash with TBST, the membrane was washed 3 times of 15 min with a denaturing washing buffer (1.5% Glycine pH2.5, 0.1% Tween-20, 1% SDS) at 95°C and 5 times of 3 min with water at room temperature, and kept at 4°C.

### srGAP2 Dot Blot

GST-tagged srGAP2 antigen was purified from BL21 *Escherichia coli* as described previously [41]. The dot blot of srGAP2 antigen was made using S&S Manifold I dot-blot array system (Sigma-Aldrich).

### Immunoblotting of the PAX Membrane

Following the PAX procedure, the peptide array membrane was re-incubated in blocking buffer for 1 h. The membrane was then cut and separated into individual bait strips and incubated with primary antibodies in the blocking buffer for I h. After extensive wash, the membrane was incubated with HRP-conjugated secondary antibodies for 1 h, washed and developed using an enhanced chemiluminescent substrate (Pierce).

### Mass Spectrometric Analysis

Following the PAX procedure, the peptide array membrane was cut and separated into bait strips. Each bait strip was further cut into small pieces and place in a Protein LoBind tube (Eppendorf), washed 3×150 uL of 50 mM ammonium bicarbonate and resuspended in 150 µl of 50 mM ammonium bicarbonate (pH8.0) containing 0.1% Rapigest (Waters, Milford MA). Samples were reduced in 10 mM dithiolthreitol at 40 C for 20 min and alkylated with iodoacetamide at 20 mM at room temperature for 40 min. On-membrane digestion was performed by adding 500 ng trypsin (Promega) and incubation at 37 C for 18 hr. Polymer contamination, mostly polyethylene glycol, were removed using ProteoSpin detergent removal spin-columns (Norgen Biotek, ON Canada, product 10200) with a modified elution buffer solution of 5% aqueous ammonia in 50 mM ammonium bicarbonate, pH 10.0). Peptides were brought to dryness using vacuum centrifugation and then resuspended in 20 uL 5% acetonitrile, 0.1% formic acid.5 uL of each sample was injected onto a Waters nanoACQUITY UPLC equipped with a 1.7-µm BEH130 C_18_ reversed-phase column [75 µm inside diameter (ID)×250 mm]. The mobile phase consisted of (A) 0.1% formic acid in water and (B) 0.1% formic acid in acetonitrile. Peptides were trapped for 5 min on a 5-µm Symmetry C_18_ column (180 µm ID×20 mm) at 20 µl/min in 99.9% A. The analytical column was then switched inline and a linear elution gradient of 5% B to 40% B was performed over 90 min at 300 nl/min. The analytical column was connected to a fused silica PicoTip emitter (New Objective) with a 10-µm tip orifice and coupled to the mass spectrometer through an electrospray interface.

MS data were acquired on an LTQ-Orbitrap XL (Thermo Scientific), mass spectrometer operating in positive-ion electrospray ionization mode. The instrument was set to acquire a precursor MS scan in the Orbitrap from mass/charge ratio (*m*/*z*) 400 to 2000 with *r* = 60,000 at *m/z* 400 and a target AGC setting of 1×10^6^ ions. In a data-dependent mode of acquisition, MS/MS spectra of either the five most abundant precursor ions were acquired in the Orbitrap at *r* = 7500 at *m/z* 400 with a target AGC setting of 2×10^5^ ions. Maximum fill times were set to 1000 ms for full MS scans and 500 ms for MS/MS scans with minimum MS/MS triggering thresholds of 1000 counts. For all experiments, fragmentation occurred in the LTQ linear ion trap with a collision-induced dissociation (CID) energy setting of 35% and a dynamic exclusion of 60 s was used for previously fragmented precursor ions.

### Qualitative Identifications from Raw LC-MS/MS Data

Raw LC-MS/MS data files were processed in Mascot distiller (Matrix Science) and then submitted to independent Mascot database searches (Matrix Science) against a Swissprot databases (*mus musculus* taxonomy) containing both forward and reverse entries of each protein. Search tolerances for LTQ-Orbitrap XL data were 20 parts per million (ppm) for precursor ions and 0.04 dalton for product ions. System suitability tests of 50 fmol of yeast alcohol dehydrogenase digest were run immediately before each sample set. All data were searched using trypsin specificity with up to two missed cleavages. Carbamidomethylation (+57.0214 daltons on C) was set as a fixed modification, whereas oxidation (+15.9949 daltons on M) was considered a variable modification. All searched spectra were imported into Scaffold (Proteome Software) and annotated using a Bayesian statistical algorithm based on the PeptideProphet and ProteinProphet algorithms, which yielded a protein FDR of 2.9%. [42], [43].

### Label-Free Quantitation of Proteins from Mouse Brain lysate

Label-free quantitation and integration of qualitative peptide identifications was performed using Rosetta Elucidator (v 3.3, Rosetta Inpharmatics, Seattle, WA). Triplicate LC-MS/MS analysis of mouse brain lysate were imported and subjected to chromatographic retention time alignment using the PeakTeller® algorithm. Quantitation of all detectable signals in the precursor MS spectra was performed within Elucidator by calculating peak height of the corresponding peptide level extracted ion chromatograms. Protein quantities were calculated using the average MS response from the two or three highest abundant peptides based on a modified strategy initially described by Silva et. al. [44]. Absolute quantities were determined by normalizing MS response factors to that of yeast alcohol dehydrogenase spiked into the lysate at 50 fmol/ug.

### Hierarchical Clustering

Relative protein abundance in the sample prepared by PAX was quantified using spectral counting [45]. We used “quantitative values” calculated within Scaffold to provide a first-pass normalization of spectral counts for each protein based on the average total spectra counts across multiple samples [46]. To further normalize the relative protein abundance, the (modified) spectral counts were expressed as a percentage of the total spectra observed in the sample. Mean normalized spectral counts were obtained from multiple independent experiments (n = 2 for each bait peptide). Hierarchical clustering was performed based on the uncentered Pearson correlation of the mean normalized spectral counts using the Cluster 3.0 program (http://bonsai.ims.utokyo.ac.jp/~mdehoon/software/cluster/) [47]. The dendrograms were viewed by the Java TreeView program (http://jtreeview.sourceforge.net/) [48].

### Bioinformatics Analysis

The known (physical) interactions were searched using Ingenuity (http://www.ingenuity.com/), GeneMANIA (version 2.0) (http://genemania.org/) and String (version 8.3) (http://string-db.org/) [49].

### Immunoprecipitation and Pull-down Experiments

Co-immunoprecipitation and pull-down experiments were performed as previously described in detail [21], [22]. Briefly, lysates were pre-cleared before incubation with antibody and either agarose conjugated protein-A or G overnight at 4 degrees C with rocking. Antibodies and bound proteins from extracts were precipitated by centrifugation and extensively washed. Bound proteins were eluted in SDS sample buffer. For GST pull-down assays, pre-cleared cell lysates were incubated with GST proteins bound to glutathione-sepharose (GE Healthcare) followed by washing, elution in sample buffer, and immunoblotting.

### Immunostaining of Cultured Mouse Fibroblast Cells

MV^D7^ cells [40] expressing EGFP-Enah were maintained as previously described. Cells were grown on a coated coverslip, fixed, stained with anti-WAVE1 antibody and imaged as described [50].
